# Supplementary material for: Geographic Mosaic of Extensive Genetic Variations in Subterranean Mole Voles Ellobius alaicus as a Consequence of Habitat Fragmentation and Hybridization
Source: Life (Basel). 2022 May 13;12(5):728. doi: 10.3390/life12050728 (PMC9146075; doi:10.3390/life12050728)
Supplement: Supplementary file 1 [file life-12-00728-s001.zip › Table S1.pdf]

Table S1. Material, localities and GenBank accession numbers.

Locality No	Species	Voucher	2n	Rb	Sex	Locality	Coordinates	GenBank accession numbers			
								cytb	*IRBP*	*XIST*, first fragment	*XIST*, second fragment
1	Possibly, backcrosses of *E. tancrei* and *E. alaicus*	27488	52	2 Rb (2.11)	f	Kyrgyzstan, Pamir highway from Osh to Gulcha (between Gulcha and Tashkoro)	N 40.25278 E 73.31554	**ON333901**	**ON333848**	**ON314941**	**ON314885**
	Possibly, backcrosses of *E. tancrei* and *E. alaicus*	27491	53	1 Rb (2.11)	f			**ON333902**	**ON333849**	**ON314942**	**ON314886**
	Possibly, backcrosses of *E. tancrei* and *E. alaicus*	27497	52	2 Rb (2.11)	m			**ON333903**	**ON333850**	**ON314943**	**ON314887**
	Possibly, backcrosses of *E. tancrei* and *E. alaicus*	27498	52	2 Rb (2.11)	m			**ON333904**	**ON333851**	**ON314944**	**ON314888**
2	*E. alaicus*	S-132131	-	-	m	Kyrgyzstan, the Taldyk pass, point 1	N 39.76667 E 73.16667	**MG264319** [1]			
	E. alaicus	S-132133	-	-	f			**MG264320** [1]			
	E. alaicus	S-132135	-	-	f			**MG264321** [1]			
3	*E. alaicus*	27354	52	2 Rb (2.11)	f	Kyrgyzstan, the Taldyk pass, point 2	N 39.74833 E 73.22766	**ON333905**	**ON333852**	**ON314945**	**ON314889**
	E. alaicus	27494	52	2 Rb (2.11)	m			**ON333906**	**ON333853**	**ON314946**	**ON314890**
4	*E. alaicus*	27351	52	2 Rb (2.11)	m	Kyrgyzstan, Sary-Tash	N 39.72392 E 73.24677	**ON333907**	**ON333854**	**ON314947**	**ON314891**
	E. alaicus	27353	52	2 Rb (2.11)	m			**ON333908**	**ON333855**	**ON314948**	**ON314892**
	E. alaicus	27501	52	2 Rb (2.11)	m						
	E. alaicus	27502	52	2 Rb (2.11)	m						
	E. alaicus	27504	52	2 Rb (2.11)	f						
	E. alaicus	27505	52	2 Rb (2.11)	m			**ON333909**	**ON333856**	**ON314949**	**ON314893**
	E. alaicus	27506	52	2 Rb (2.11)	m						
5	*E. alaicus*	27489	52	2 Rb (2.11)	f	Kyrgyzstan, Taunmuruk vicinities	N 39.64459 E 73.76935	**ON333910**	**ON333857**	**ON314950**	**ON314894**
	E. alaicus	27495	52	2 Rb (2.11)	m			**ON333911**	**ON333858**	**ON314951**	**ON314895**

Locality No	Species	Voucher	2n	Rb	Sex	Locality	Coordinates	GenBank accession numbers			
								cytb	*IRBP*	*XIST*, first fragment	*XIST*, second fragment
6	*E. alaicus*	27496	52	2 Rb (2.11)	m	Kyrgyzstan, about 3 km to the west from Sary-Tash	N 39.7029 E 73.21874	**ON333912**	**ON333859**	**ON314952**	**ON314896**
7	*E. alaicus*	27493	51	2 Rb (2.11) 1 Rb (3.10)	m	Kyrgyzstan, Sary-Mogol vicinities	N 39.619 E 72.60924	**ON333913**	**ON333860**	**ON314953**	**ON314897**
	E. alaicus	27500	50	2 Rb (2.11) 2 Rb (3.10)	m			**ON333914**	**ON333861**	**ON314954**	**ON314898**
	E. alaicus	27503	50	2 Rb (2.11) 2 Rb (3.10)	m			**ON333915**	**ON333862**	**ON314955**	**ON314899**
8	*E. alaicus*	S-132130	-	-	m	Kyrgyzstan, Daroot-Korgon, point 1	N 39.55 E 72.25	**MG264318 [1]**			
9	Possibly, backcrosses of *E. tancrei* and *E. alaicus*	27490	50	2 Rb (2.11) 2 Rb (3.10)	f	Kyrgyzstan, Daroot-Korgon, point 2 (near bridge across Kyzylsu)	N 39.53976 E 72.1712	**ON333916**	**ON333863**	**ON314956**	**ON314900**
		27499	51	2 Rb (2.11) 1 Rb (3.10)	m			**ON333917**	**ON333864**	**ON314957**	**ON314901**
10	*E. alaicus*	27487	50-51	2 Rb (2.11) 1-2 Rb (3.10)	m	Kyrgyzstan, Daroot-Korgon, point 3	N 39.53918 E 72.17201	**ON333918**	**ON333865**	**ON314958**	**ON314902**
	E. alaicus	27492	51	2 Rb (2.11) 1 Rb (3.10)	m			**ON333919**	**ON333866**	**ON314959**	**ON314903**
11	*E. alaicus*	25605	48	2 Rb (2.11), 2 Rb (4.9), 2 Rb (3.10)	f	Tajikistan, Achek-Alma	N 39.37883 E 71.678	**MG264322 [1]**	**ON333867**	**MK544925 [1]**	**ON314904**
	E. alaicus	25611	48	2 Rb (2.11), 2 Rb (4.9), 2 Rb (3.10)	m			**MG264324 [1]**	**ON333868**	**ON314960**	**ON314905**
12	*E. alaicus*	27025	48	2 Rb (2.11), 2 Rb (4.9), 2 Rb (3.10)	m	Tajikistan, Dzhailgan	N 39.32128 E 71.54537	**MK544910 [1]**	**ON333869**	**MK544926 [1]**	**ON314906**
	E. alaicus	27026	48	2 Rb (2.11), 2 Rb (4.9), 2 Rb (3.10)	m			**MK544911 [1]**	**ON333870**	**ON314961**	**ON314907**

Locality No	Species	Voucher	2n	Rb	Sex	Locality	Coordinates	GenBank accession numbers			
								cytb	*IRBP*	*XIST*, first fragment	*XIST*, second fragment
13	*E. alaicus*	25602	48	2 Rb (2.11), 2 Rb (4.9), 2 Rb (3.10)	f	Tajikistan, Duvana	N 39.345 E 71.57883	**MG264326** [1]	**ON333871**	**MK544924** [1]	**ON314908**
14	*E. alaicus*	27028	48	2 Rb (2.11), 2 Rb (4.9), 2 Rb (3.10)	f	Tajikistan, Kashat vicinities, near bridge across Kyzylsu	N 39.30748 E 71.47467	**MK544913** [1]	**ON333872**	**ON314962**	**ON314909**
	E. alaicus	27029	48	2 Rb (2.11), 2 Rb (4.9), 2 Rb (3.10)	f			**MK544914** [1]	**ON333873**	**ON314963**	**ON314910**
15	*E. alaicus*	27030	48	2 Rb (2.11), 2 Rb (4.9), 2 Rb (3.10)	f	Tajikistan, Muksu left bank	N 39.2458 E 71.41667	**MK544915** [1]	**ON333874**	**ON314964**	**ON314911**
	E. alaicus	27031	48	2 Rb (2.11), 2 Rb (4.9), 2 Rb (3.10)	f			**ON333920**	**ON333875**	**ON314965**	**ON314912**
	E. alaicus	27032	48	2 Rb (2.11), 2 Rb (4.9), 2 Rb (3.10)	f			**MK544916** [1]	**ON333876**	**ON314966**	**ON314913**
	E. alaicus	27033	48	2 Rb (2.11), 2 Rb (4.9), 2 Rb (3.10)	m			**MK544917** [1]	**ON333877**	**ON314967**	**ON314914**
16	*E. tancrei*	27019	54	no Rbs	m	Tajikistan, Utol-Poyon	N 39.16228 E 71.1229	**MK544906** [1]	**MT478770** [2]	**ON314968**	**ON314915**
	E. tancrei	27020	54	no Rbs	f			**MK544907** [1]	**ON333878**	**ON314969**	**ON314916**
	E. tancrei	27021	54	no Rbs	m			**MK544908** [1]	**ON333879**	**ON314970**	**ON314917**
	E. tancrei	27022	54	no Rbs	f			**MK544909** [1]	**ON333880**	**ON314971**	**ON314918**
17	*E. tancrei*	27017	54	no Rbs	m	Tajikistan, between Kichikzy and Utol-Poyon	N 39.12708 E 70.99603	**MK544904** [1]	**ON333881**	**MK544923** [1]	**ON314919**
	E. tancrei	27027	54	no Rbs	m			**MK544912** [1]	**ON333882**	**ON314972**	**ON314920**
18	*E. tancrei*	24898	52	see [3]	m	Tajikistan, Kichikzy	N 39.823 E 70.5733	**ON333921**	**ON333883**	**ON314973**	**ON314921**
	E. tancrei	24899	51	see [3]	f			**ON333922**	**ON333884**	**ON314974**	**ON314922**
19	*E. tancrei*	25604	32	see [3]	m	Tajikistan, Sarinai	N 39.0645 E 70.8716	**ON333923**	**ON333885**	**ON314975**	**ON314923**
	E. tancrei	25613	32	see [3]	m			**ON333924**	**ON333886**	**ON314976**	**ON314924**

Locality No	Species	Voucher	2n	Rb	Sex	Locality	Coordinates	GenBank accession numbers			
								cytb	*IRBP*	*XIST*, first fragment	*XIST*, second fragment
20	*E. tancrei*	25606	32	see [3]	m	Tajikistan, Obi-Kaboud	N 39.22527 E 70.85218	**ON333925**	**ON333887**	**ON314977**	**ON314925**
21	*E. tancrei*	25601	30	see [4]	f	Tajikistan, Shilbili	N 39.25617 E 71.34317	**MG264327** [1]	**ON333888**	**ON314978**	**ON314926**
	E. tancrei	25618	30	see [4]	f			**MG264328** [1]	**ON333889**	**ON314979**	**ON314927**
	E. tancrei	25625	30	see [4]	m			**MG264329** [1]	**ON333890**	**ON314980**	**ON314928**
	E. tancrei	25626	30	see [4]	f			**MG264330** [1]	**ON333891**	**ON314981**	**ON314929**
22	*E. tancrei*	25603	34	see [3]	m	Tajikistan, Khozar-Chashma	N 39.03083 E 70.50472	**ON333926**	**ON333892**	**ON314982**	**ON314930**
	E. tancrei	25617	34	see [3]	m			**ON333927**	**ON333893**	**ON314983**	**ON314931**
23	*E. tancrei*	25608	34	see [3]	m	Tajikistan, Saripoul	N 38.8775 E 70.0007	**ON333928**	**ON333894**	**ON314984**	**ON314932**
	E. tancrei	25609	34	see [3]	f			**ON333929**	**ON333895**	**ON314985**	**ON314933**
24	*E. tancrei*	24913	54	no Rbs	m	Tajikistan, Romit	N 38.75455 E 69.29332	**MG264345** [1]	**MT478769** [2]	**MK544921** [1]	**ON314934**
25	*E. tancrei*	27016	54	no Rbs	m	Tajikistan, Sovetabad	N 37.47465 E 68.25947	**MK544903** [1]	**ON333896**	**ON314986**	**ON314935**
26	*E. tancrei*	27013	54	no Rbs	m	Tajikistan, Aivadj	N 36.96947 E 68.01152	**MK544901** [1]	**ON333897**	**ON314987**	**ON314936**
27	*E. tancrei*	25159	54	no Rbs	m	Uzbekistan, Tashkent	N 41.3415 E 70.31183	**MG264346** [1]	**MT478768** [2]	**MK544922** [1]	**ON314937**
28	*E. tancrei*	25255	54	no Rbs	-	Mongolia, Bulgan	N 46.95417 E 91.19139	**MT468380** [2]	**ON333898**	**ON314988**	**ON314938**
29	*E. tancrei*	25266	54	no Rbs	f	Mongolia, Khargantyn Ula	N 49.15428 E 89.92281	**ON333930**	**ON333899**	**ON314989**	**ON314939**
30	*E. tancrei*	26721	54	no Rbs	f	Mongolia, Arakhangai	N 47.347500, E 101.844444	**ON333931**	**ON333900**	**ON314990**	**ON314940**

Notes: [1] – cited in [18], [2] – cited in [14].
