# Supplementary material for: Geographic Mosaic of Extensive Genetic Variations in Subterranean Mole Voles Ellobius alaicus as a Consequence of Habitat Fragmentation and Hybridization
Source: Life (Basel). 2022 May 13;12(5):728. doi: 10.3390/life12050728 (PMC9146075; doi:10.3390/life12050728)
Supplement: Supplementary file 1 [file life-12-00728-s001.zip › Table S2.pdf]

Table S2. List of used primers for amplification and sequencing of *cytb, XIST, IRBP* genes

Gene<br>Fragment	Primers	T ºC	Source
cytb			
First fragment	Eta_cytbF1 (GAAACACCTAATGACAATCATACG)<br>MVZ04m (GTGGCCCCTCAAAATGATATTTGTCCTC)	57 ºC	[13]
Second fragment	Eta_cytbF1 (GAAACACCTAATGACAATCATACG)<br>CLETH16m (AGGAAGTACCATTCTGGTTTAAT)	57 ºC	[13]
Third fragment	Vole23m (TCCTGTTCCTTCACGAAACAGGTTC)<br>Vole14 (TTTCATTACTGGTTTACAAGAC)	55 ºC	[13]<br>[23]
IRBP, exon 1			
First fragment	F11 (CAGCCATTGAGCAGGCTATGAA)<br>R22_cric (AGACCACGGCTGAGTAGTCCAT)	63 ºC	[24]
Second fragment	IRBP2-Ftal (CAGACGTGGGAAGGCAGTGGAG)<br>IRBP2-1Rtal (GGCAAACTCCTCAGCAGCGGTAGC)	67 ºC	Present study
XIST			
First fragment	Xist1-L11841 (GGGGTCTCTGGGAACATTTT)<br>Xist1-R12504 (TGCAATAACTCACAAAACCAAC) or	63 ºC	[18]
	Xist1-L11929 (GATTTTATGTGTTCTGGTAGG)<br>Xist1-R12504 or	63 ºC	Present study
	Xist1-L11841<br>Xist1-Rint (AAGCAGGTAAGTATCCACAGC)	63 ºC	Present study
Second fragment	Xist1-L11841<br>Xist-R12995 (GGGTTGTCGAGCCTTGGGTAGAGT) or	63 ºC	Present study
	Xist2-Fint (GTGGATGGATATATGTTGGTTTTG)<br>Xist-R12995	63 ºC	Present study

Notes: sequences of forward (upper line) and reverse (bottom line) primers (5'–3') are presented for each studied gene fragment in the column "Primers". "T ºC" is annealing temperatures.
